# Effects of Silicone Rubber on Rheological Properties and Aging Characteristics of Asphalt Binder

**DOI:** 10.3390/polym16131903

**Published:** 2024-07-02

**Authors:** Maoqing Li, Zichen Gao, Zewen He, Jiachen Ma, Wenhui Zhao, Shihao Dang, Chenhao Wei

**Affiliations:** 1 Shaanxi Coal Chemical Industry Technology Research Institute Co., Ltd., Xi’an 710100, China; 2 Tianjin Transportation Research Institute, Tianjin 300060, China; 3 School of Materials Science and Engineering, Chang’an University, Xi’an 710018, China; hezewen@chd.edu.cn (Z.H.); zhaowenhui1995@126.com (W.Z.)

**Keywords:** silicone rubber, rheological properties, high-temperature stability, toluene insoluble test

## Abstract

Silicone rubber (SR) is a kind of polymer insulation material with excellent performance. With the service life of silicone rubber products reaching the limit, how to dispose of waste silicone rubber is an urgent problem to be solved. In this paper, silicone rubber-modified asphalt binder (SRMA) was prepared by SR and 90# base asphalt binder. The simulated short-term aging and long-term aging tests of SRMA were carried out using the thin film oven aging test (TFOT) and pressure aging vessel test (PAV). The rotary viscosity test and dynamic shear rheological test (DSR) were applied to the rheological properties of SRMA before and after aging. The degradation degree and chemical composition changes of SR were explored by the toluene insoluble matter test, Fourier transform infrared spectroscopy (FTIR), and a Fluorescence microscope (FM). The results demonstrate that SR can significantly affect the aging resistance, fatigue life, and high-temperature stability of SRMA. As the content of SR rose, the elastic component in SRMA increased, leading to a nice performance in stability at high temperatures and fatigue resistance. However, excessive content (14%wt and 16%wt) had a negative influence on the performance of SRMA. So, the optimal content was speculated to be between 12% and 14%. Furthermore, SR and asphalt binder would be aged and degraded together in the aging process, and this phenomenon was more obvious during long-term aging.

## 1. Introduction

In recent years, transmission line projects have advanced quickly. As the main material of insulators, silicone rubber is widely used in industry. The lifespan of silicone rubber insulators typically exceeds 15 years. However, in China, over half of the insulators currently in use have been in service for more than 10 years. This indicates that a significant number of silicone rubber insulators will soon be discarded. What is more, wastes like structured silicone rubber and cooked rubber are generated during rubber mixing, and defective products and scraps are also inevitably generated. According to the calculation of the production capacity of China’s composite insulator production enterprises, the annual output of composite insulators produces more than 2000 tons of waste silicone rubber [1,2,3]. However, they were piled up due to their difficulty in degrading, which caused resource waste and environmental pollution. Therefore, the proper disposal of discarded SR has become an urgent problem to be solved.

There are two main methods for treating discarded SR in recent research, including the pulverization method and pyrolysis [1,3]. The former uses crushed SR as an organic resin, thermoplastic elastomers, coatings, inks, and construction materials. The latter converts waste SR into rubber row materials suitable for further processing, vulcanization, and siloxane monomer. However, both methods have low SR utilization, complicated operations, and huge energy consumption, which cannot fundamentally solve the problem. Thus, some academics discussed whether it could be used in the same way that other rubbers were, while building roads.

Rubber-modified asphalt binder is a composite material composed of the full swelling reaction of rubber powder and base asphalt binder at high temperatures. It has been widely used in road construction in various countries because of its excellent high-temperature properties [4,5,6]. According to statistics, rubber asphalt binder pavements have a service life that is 2–5 years longer than conventional asphalt binder pavements and are superior at resisting rutting [7,8]. Numerous studies have indicated that crumb rubber (CR) can lower permeability and ductility as well as raise the softening point and viscosity [9] and greatly boost aging resistance [10,11,12]. This was demonstrated by Wang et al. [13] in a dynamic shear rheology test (DSR). The complex modulus, phase angle, unrecoverable creep compliance, and other rheological parameters changed in CRMA after aging more slowly than in base asphalt binder, which was consistent with CRMA aging with lower carbonyl and sulfoxide indices [14]. Meanwhile, scholars discovered in microscopic research that the aging of polymer modified asphalt binder (PMA) was a complex process that included not only the aging of base asphalt binder and deterioration of polymers but also their interactions during aging [15,16], and both the time and temperature of the interactions could affect properties of CRMA [17]. The internal CRMA undergoes an intense swelling reaction during the short-term aging (STA) process, and the base asphalt binder wraps around CR to protect it from oxygen [18] and reduce CR degradation. In the meantime, CR absorbs the lighter components and continuously dissolves into the asphalt phase [19,20], improving CRMA aging resistance. With the deepening of aging, the polymer grid was destroyed, and the wrapping of asphalt binder on CR became worse, resulting in the degree of blending and the temperature sensitivity being reduced [21].

Inspired by CRMA, Shen [22] et al. modified asphalt binder with waste SR. It has been demonstrated through basic index tests that SR can improve the softening point and ductility, reduce penetration, and improve high-temperature performance. T.M. Ai-ani [23] and Masoud Fakroun [24] investigated the properties of the SR-modified asphalt mixture. Marshall tests reveal that SR can increase Marshall stability and reduce bulk density, which makes the application of SR in modified asphalt possible. In summary, although many researchers have explored the aging of CRMA in the current research, the research on SR was limited to the effects of SR content, particle size, and mixing temperature on the performance of SRMA at high or low temperatures, and research into its aging performance remained incomplete.

**Figure 1:**
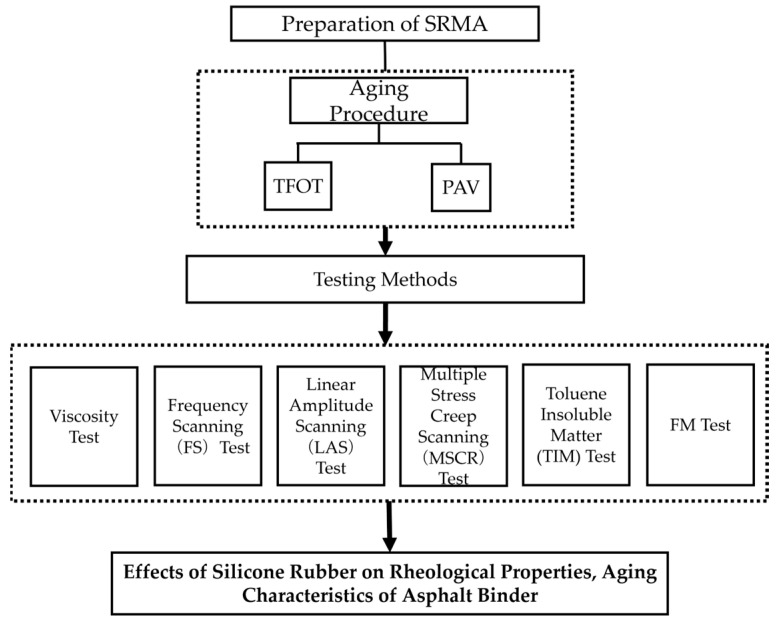
Research program.

The effects of silicone rubber on asphalt properties before and after aging were studied. The aging process of asphalt in practical applications was simulated by the thin film oven test (TFOT) and pressure Aging Vessel Test (PAV). The rheological properties of aged SRMA are evaluated by Brinell rotational viscometer, frequency scanning (FS), linear amplitude scanning (LAS), and MSCR. Additionally, the toluene insoluble test (TIM), Fourier transform infrared spectroscopy (FTIR), and a fluorescence microscope (FM) were applied to observe the physical and chemical changes of silicone rubber in asphalt and the interaction between rubber particles and asphalt molecules at different aging conditions. The content route of this study is shown in Figure 1.

## 2. Materials and Methods

### 2.1. Row Materials

**Figure 2:**
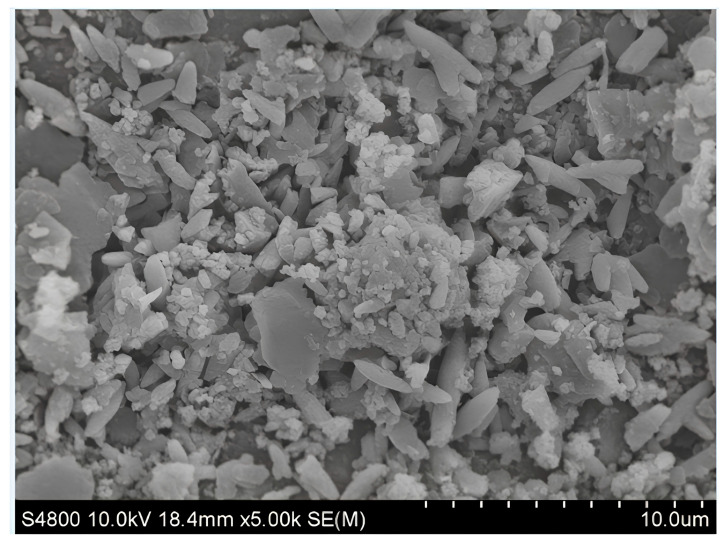
The micromorphology of SR.

In this research, 90#Donghai asphalt and 100-mesh SR powder were used, where the SR was provided by Hengtai Plastic Co., Ltd. The basic physical properties according to JTG E20-2011 [25] are shown in Table 1 and Table 2, and the micromorphology of SR is shown in Figure 2. According to the SEM of silicone rubber powder, it can be seen that it is mainly cylindrical and flaky debris with relatively flat surface. Silicone rubber is a linear organic siloxane elastomer material. The side chain groups of silicone rubber used in this study are mainly methyl and vinyl, so it is more heat resistant than traditional rubber.

It is found that when SR is too fine, it is easy to agglomerate in the asphalt, resulting in SR that cannot be uniformly dispersed in the asphalt, which will worsen the modification effect. If the SR is too coarse, it will increase the difficulty of the mixing and shearing process of modified asphalt.

### 2.2. Preparation of SRMA

A certain amount of dried SR powder (8%,10%,12%,14%,16%by the mass of asphalt) was mixed with the base asphalt binder at 180 °C and agitated in a shear mixer at 5000 r/min shear rate for 1 h. The preparation parameters used in this study are based on the common application of polymer modified asphalt in practical engineering. The mixture was then expanded at 160 °C for 40 min to expel air by manual stirring. Finally, the prepared SRMA was poured into two aging plates, which are 50 g each, for subsequent aging experiments. When the temperature reaches 180 °C, the dispersion uniformity of silicone rubber in asphalt can be ensured, and the construction temperature of the asphalt mixture is consistent. The choice of 5000 r/min and 1 h is due to the need to fully shear and stir the silicone rubber.

### 2.3. Aging Procedure

For the SRMA short-term aging (STA) procedure, a thin film oven test (TFOT) was carried out at 163 °C for 5 h on the prepared aging plates containing 50 g of sample referred to ASTM D1754. Furthermore, according to ASTM D6521, the sample after STA was subjected to a long-term aging test (LTA) in a pressurized aging vessel (PAV) for 20 h at a temperature of 100 °C and 2.1 MPa. TFOT can simulate short-term aging during asphalt preparation, while PAV can simulate the aging of asphalt pavement after long-term service.

### 2.4. Testing Methods

#### 2.4.1. Viscosity Test

The viscosity of SRMA was tested at 180 °C according to ASTM D4402. The sheer speed of 20 r/min was adopted to result in a torque in the range of 10% to 98%.

#### 2.4.2. Frequency Scanning (FS) Test

The linear viscoelastic parameters of the asphalt materials were determined by the FS test. Frequency scanning can simulate the deformation resistance of asphalt at different speeds. According to the time–temperature equivalence principle, frequency scanning can reflect the deformation resistance of asphalt at high temperature and low frequency or low temperature and high frequency. In this paper, the complex shear modulus (G*) and phase angle (δ) of SRMA before and after aging were obtained by using a single temperature of 64 °C and loading frequency from 100 Hz to 1 Hz to further obtain the rutting factor (G*/sin δ), which was defined as the rutting resistance factor of asphalt binder in order to assess its permanent high-temperature strain [10].

#### 2.4.3. Linear Amplitude Scanning (LAS) Test

Based on the AASHTO TP 101, the LAS test at 25 °C was accomplished at 10 Hz with an increase in the magnitude of the strain from 0.1% to 30%. The failure strain and the strain dependence of SRMA can be analyzed through the stress-strain curve. In addition, the simplified viscoelastic continuum damage (S-VECD) model was applied to predict the fatigue characteristics of SRMA before and after aging [26].

#### 2.4.4. Multiple Stress Creep Scanning (MSCR) Test

MSCR test mainly reflects the stress and strain characteristics of asphalt binder under different constant stresses. After the stress is removed, part of the end strain will recover, and the unrecoverable strain will be accumulated in the next load cycle, which can truly simulate the repetitive loading and unloading of pavement by vehicles and can effectively assess asphalt binder performance at high temperatures. The delayed elastic recovery performance of SRMA was evaluated by recovery percentage (R) and unrecoverable creep compliance (J_nr_) [27], as shown in Equations (1) and (2).

According to ASTM D7405, the MSCR test temperature was 64 °C, loading stress was 0.1 kPa and 3.2 kPa, 1 s were loaded, and 9 s were unloaded in a creep recovery cycle for a total of 10 cycles. R represents the deformation recovery rate; Jnr stands for non-recoverable creep compliance. The average of 10 cycles at two stress levels was used as an evaluation indicator, represented by R0.1, R3.2, J_nr0.1_, and J_nr3.2_, respectively.

(1) $$R=\frac{\varepsilon_{p}-\varepsilon_{u}}{\varepsilon_{p}}$$

(2) $$J_{nr}=\frac{\varepsilon_{u}}{\sigma }$$

where $\varepsilon_{p}$ is the peak strain; $\varepsilon_{u}$ is the unrecovered strain; σ is the creep stress.

#### 2.4.5. Toluene Insoluble Matter (TIM) Test

Depending on the insoluble content of SR in toluene, the degree of SR degradation in aged SRMA could be analyzed and the remaining content of SR could be estimated quantitatively [28,29,30]. The experimental procedure was as follows: first, 0.5 g of SRMA(M_0_) was wrapped with medium-speed filter paper and the mass (M_1_) was weighed; then, it was placed in the extractor, which connects the flat-bottomed flask containing 40 mL of toluene and a spherical condenser tube. The entire apparatus was placed in the heating jacket and heated to 150 so that the refluxed toluene continuously washes the SRMA until the filtrate was colorless. The filter paper containing insoluble material was finally placed in an oven at 70 °C, and the dried mass (M_2_) was weighed. The TIM content (TC) and attenuation rate of TC (Q) were calculated according to Equations (3) and (4) [31]; the TIM content of the pure SR powder was found to be 83.00% (TC_0_).

(3) $$TC=\frac{M_{2}-M_{1}+M_{0}}{M_{0}\times SRcontent}$$

(4) $$Q=\frac{TC_{0}-TC}{TC_{0}}\times 100\%$$

#### 2.4.6. FTIR Test

Fourier transform infrared spectroscopy (FTIR) was used to investigate SRMA functional groups before and after aging in the 500 to 3500 cm^−1^ wavelength range, and spectra were acquired at 4 cm^−1^ resolution.

#### 2.4.7. FM Test

A fluorescence microscope (YG-100) was used to observe the dispersion of the polymer in PMA. A tiny drop of bitumen was attached to the heated slide and pressed with a coverslip to form a thin sample for the FM test.

## 3. Results and Discussion

### 3.1. Viscosity

**Figure 3:**
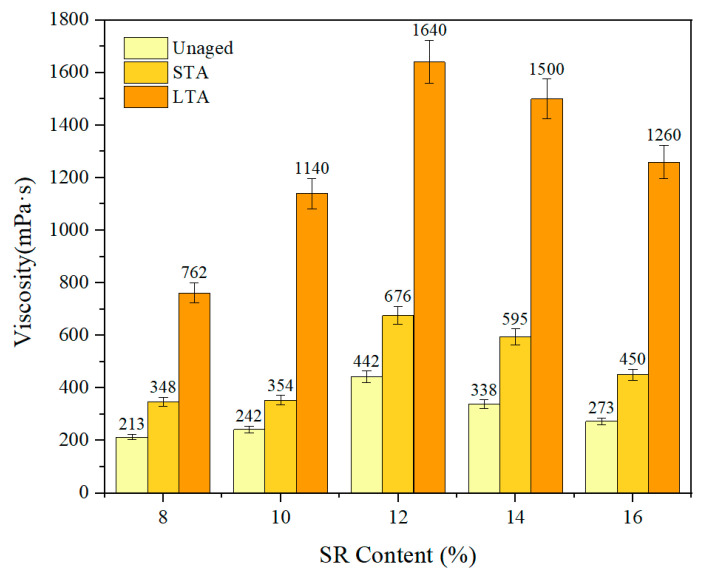
The Viscosity of SRMA at 180 °C.

The viscosity of SRMA with different contents before and after aging is shown in Figure 3. As expected, the viscosity of asphalt binder increased significantly with the SR content increased due to the SR absorbing the light components after blending with asphalt binder, which will increase the proportion of asphaltene ratio and make the asphalt binder viscous [9]. Further, the viscosity of SRMA peaked at 12% and then decreased, but it was still higher than that of SRMA with low content (8%), which may be related to the agglomeration and segregation of SR with high content because the phenomenon of SR agglomeration and floating can be observed intuitively during the viscosity test. When the content of silicone rubber exceeds 14%, SR is prone to agglomeration, and SR cannot fully swell and uniformly disperse in the asphalt. Therefore, the aggregation and segregation of SR leads to insufficient swelling of silicone rubber and uneven interior of the modified asphalt, resulting in reduced viscosity

Compared to SRMA with different aging degrees at the same content, the viscosity of SRMA increased rapidly with the deepening of aging. For example, the viscosity of 12% increased by only 52.9% after STA but increased sharply to 271% after LTA, as well as for 14% SRMA, which increased by 76% and 344% in the two aging degrees. Because there was a strong swelling process of SR in SRMA during STA, which caused the structure of rubber molecules to relax [18]. Some of the smaller polymer molecules in the rubber melt into asphalt binder, and aging caused partial degradation of the polymer, leading to further chain breaks dissolved in the asphalt phase, which hindered the increase in viscosity, resulting in a slow increase in viscosity [32,33]. In the LTA stage, the continuous rubber swelling effect of rubber added in asphalt was greatly weakened, and the asphalt phase continued to oxidize, resulting in the rapid increase in viscosity.

### 3.2. FS

#### 3.2.1. Test Results of Complex Shear Modulus

**Figure 4:**
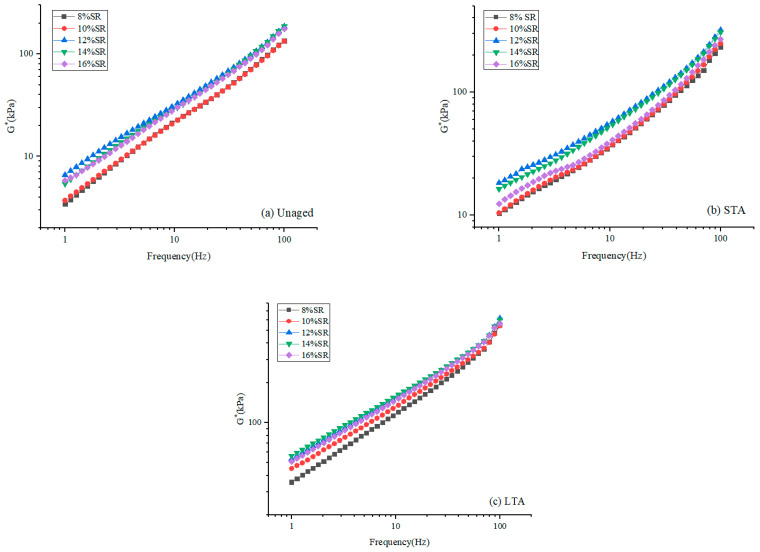
The complex modulus G* of SRMA with different content: (**a**) Unaged; (**b**) STA; (**c**) LTA.

For viscoelastic materials, the stress-strain ratio was a complex shear modulus (G*), which may reflect the ability of SRMA to withstand shear strain to some degree [34]. Figure 4 shows the complex modulus G* test results of SRMA with different content under different aging degrees. Clearly, aging caused the SRMA master curve with five contents to shift upward, pointing to a common trend of asphalt binder gradually hardening with deepening age. In addition, the high content of SRMA showed the characteristics of “low frequency and high elasticity”, and the characteristics of SRMA with a content of 12% and 14% were more obvious.

#### 3.2.2. Test Results of Phase Angle

The phase angle(δ) provided information on the ratio between the elastic and viscous responses of the SRMA. In general, δ the smaller, the closer the SRMA was to the elastomer [34].

**Figure 5:**
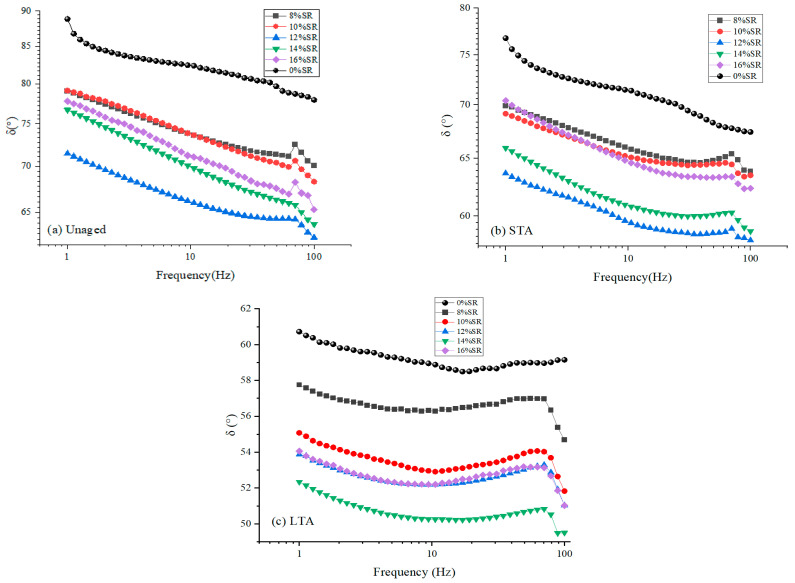
The phase angle (δ) of SRMA with different content: (**a**) Unaged; (**b**) STA; (**c**) LTA.

The phase angle test results of SRMA with different content under different aging degrees are shown in Figure 5. As expected, with the increase in SR content, the elastic component in SRMA increased and δ decreased, but the abnormal phenomenon still occurred in SRMA with 16% SR content (i.e., SRMA with phase angle greater than 12% and 14% doped). In addition, the effect of aging can also increase the proportion of the elastic part, thus reducing δ. Surprisingly, δ changed when the frequency was 70 Hz. For polymer modified asphalt, the greater the frequency of frequency scanning, the more obvious the elastic performance of polymer modified asphalt, so the phase angle gradually decreases. However, when the frequency increases to a certain extent, such as 70 Hz in this study, the phase angle of silicone rubber-modified asphalt will mutate.

#### 3.2.3. Test Results of Rutting Factor (G*/sin δ)

The high-temperature rutting resistance of SRMA was reflected in the rutting factor (G*/sin δ). The rutting factor can reflect the high-temperature rutting resistance of asphalt more directly. The higher the frequency, the larger the rutting factor and the better the rutting resistance of asphalt. The higher the load, the lower the rutting factor and the worse the rutting resistance. As the G*/sin δ increases, the binder flow strain decreases and the binder rutting resistance increases as temperature rises. With the increase in loading frequency, the G*/sinδ increased at a certain temperature because higher frequencies represented faster driving speed; the load acted for a short time and dissipated before it could spread [35].

**Figure 6:**
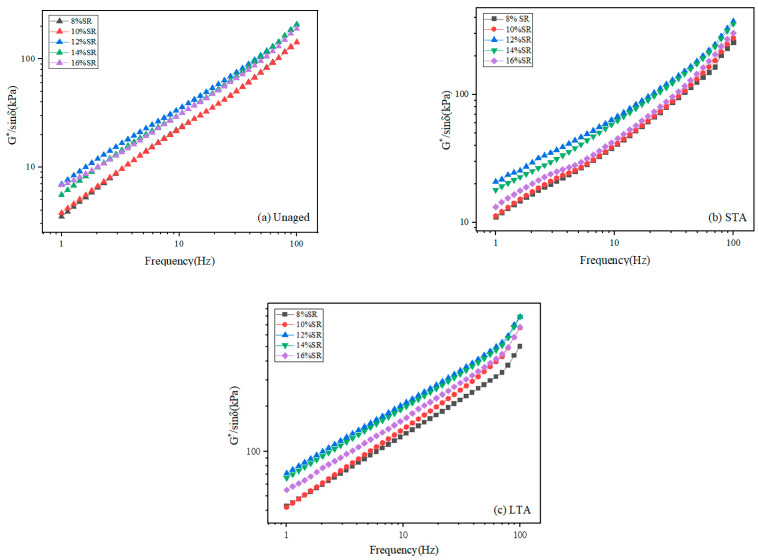
The rutting factor (G*/sin δ) of SRMA with different content: (**a**) Unaged; (**b**) STA; (**c**) LTA.

The trends of the rutting factor of SRMA with different content under different aging degrees are shown in Figure 6a–c. The G*/sin δ values of SRMAs (over 12% SR) were generally greater than those of SRMAs (below 10% SR), especially in the low-frequency range. In addition, the rutting factors of 14% SRMA before and after aging were very close to the 12% SRMA; they were both greater than SRMA with 16% content, indicating that a certain amount of SR can significantly improve the high-temperature permanent strain resistance of asphalt binder [36], and there was an optimal content of SR. Compared with the unaged SRMA in Figure 6a, the G*/sin δ of SRMA after aging was greater than the unaged with the aging degree of SRMA gradually deepened, indicating that aged SRMA had better rutting resistance.

### 3.3. LAS

**Figure 7:**
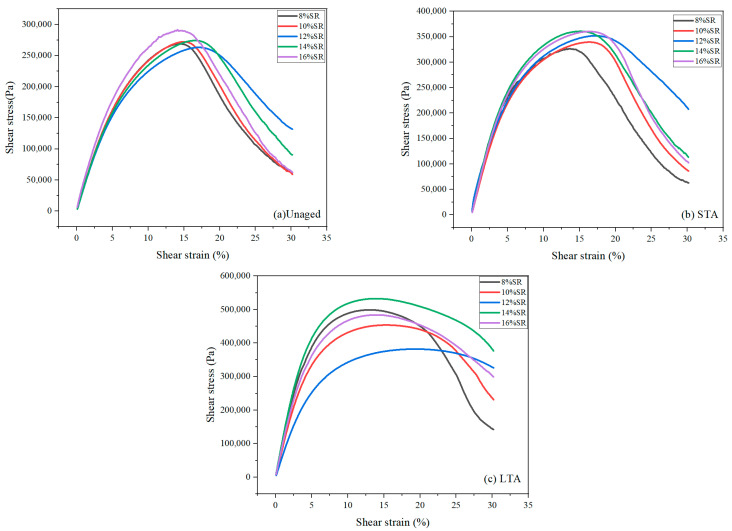
The stress-strain diagram of SRMA with different content: (**a**) Unaged; (**b**) STA; (**c**) LTA.

LAS was the main test method to obtain the linear viscoelastic parameters of asphalt materials. The failure strain of SRMA could be obtained via the curves in Figure 7, and the dependence of material properties on strain could be analyzed. The width of the curve at the peak represented the strain dependence of the material [25]. The stress-strain diagram of SRMA with different content under different aging conditions is shown in Figure 7. It was clear that with increased SR content, there was an increasing trend in the width of the peak of the curves, indicating that SRMA with high content had a higher dependence on the corresponding strain.

**Figure 8:**
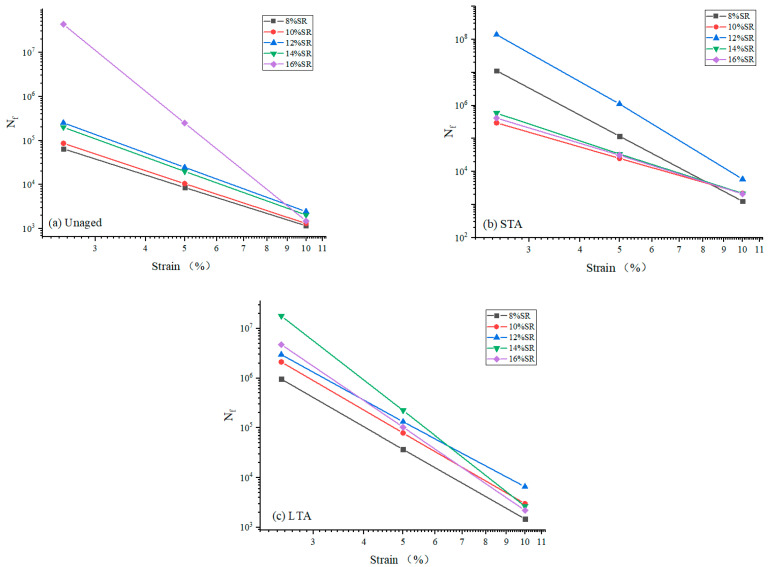
The fatigue life (N_f_) of SRMA with different content: (**a**) Unaged; (**b**) STA; (**c**) LTA.

In addition, the S-VECD model was used to make a simple prediction of the fatigue life (N_f_) of the SRMA, and the changing trend of SRMA N_f_ at different strains (2.5%, 5%, and 10%) is shown in Figure 8. With the increase in SR content, the N_f_ of SRMA increased gradually, and the performance of SRMA was the most stable when the content was 12%, which was slightly higher than that of SRMA with 14% and 16% content before and after aging, proved that a certain amount of SR can better improve the fatigue resistance of asphalt binder. After a short-term aging process, the level of SR in asphalt directly correlates to the absorption of light components, ultimately impacting the degree of asphalt aging and subsequently reducing its fatigue life. In contrast, during long-term aging, the resilience of asphalt against weathering is determined by the quantity of SR present. A higher SR content results in less degradation due to aging, thereby enhancing the fatigue resistance of asphalt materials over extended periods.

### 3.4. MSCR

PMA has a certain delayed elastic recovery performance. Below a certain recovery time, the greater the recovery elastic strain in the total strain, the smaller the amount of unrecoverable strain, and the lower the incidence of rutting was [37]. R in MSCR can reflect the elastic recovery ability of asphalt pavement after stress. The higher the value of R, the stronger the recovery ability of asphalt, and the less rutting disease is likely to occur under high-temperature conditions. Jnr can reflect the non-recoverable composition of asphalt.

**Figure 9:**
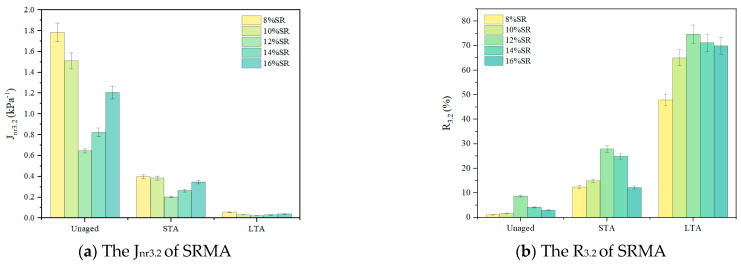
The J_nr3.2_ and R_3.2_ of SRMA with different content.

R at the loading level of 0.1 kPa and 3.2 kPa had a similar law, and the law was more obvious at the load level of 3.2 kPa, as well as for Jnr. Therefore, R and Jnr at the load level of 3.2 kPa are shown in Figure 8 and Figure 9. Obviously, the partial creep strain of all SRMA had recovered at the end of each cycle. With the increase in SR content, the elastic component in SRMA increased, and the recovery degree after creep increased. When the content exceeded 12%, the degree of recovery declined, but it was still higher than that of low-content SRMA (8%), which proved that the incorporation of SR can significantly improve the elastic recovery performance of SRMA at high temperatures.

In addition, the SRMA with different aging degrees under the same content showed that after STA and LTA, the R_3.2_ of SRMA was improved to varying degrees (opposite to the low of J_nr_). For example, R3.2 of SRMA with 12% content increased slightly from 8.75% to 27.93% after STA and rapidly increased to 74.73% after LTA, while SRMA with 14% content increased from 4.2% to 24.95% and 71.21% respectively. It was well known that the polymer degradation of PMA during aging will reduce the elastic recovery, while the hardening of the asphalt phase during aging will improve the elastic recovery [27]. Due to the oxygen barrier protection of SRMA in the actual aging process of SRMA [18], the degradation of SR was limited, resulting in the hardening of the asphalt phase after aging, which played a major role in affecting the elastic recovery performance of SRMA. Therefore, under the same content, the recovery degree of SRMA after aging was higher than that of unaged SRMA, and LTA was also better than STA.

### 3.5. Toluene Insoluble Matter

**Figure 10:**
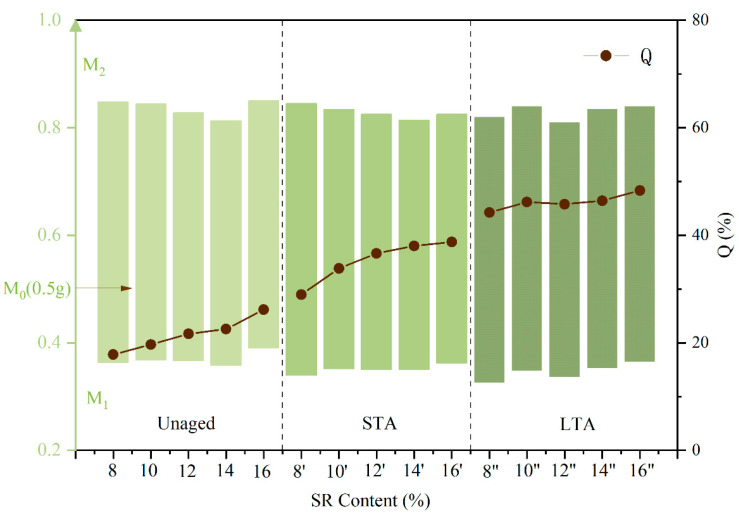
The attenuation rate (Q) of SRMA with different content under different aging degrees.

SR powder can be decomposed into small molecules or toluene-soluble substances after high-temperature shearing and aging. The rate of attenuation (Q) reflects the severity of the SR chain interruption reaction [38]. The Q value of SRMA with different content under different aging degrees is shown in Figure 10. The green rectangle in the figure represents the modified asphalt with different SR content according to the quality difference before and after toluene dissolution in the test process for toluene insoluble matter. The top edge of the rectangle represents the quality of asphalt to be measured before dissolution, and the bottom edge represents the quality of insoluble matter after dissolution.

The Q values at different aging stages exhibit a consistent trend, gradually increasing with the rise in SR content. Substantial changes in Q value are observed after short-term and long-term aging. As the asphalt aging degree worsened, there was an evident increase in small molecule substances following SR chain fracture and degradation, leading to a decrease in toluene insoluble content and a notable rise in Q value. When the SR content reaches 8%, the Q value can increase by 16% after long-term aging, marking the largest increase during this period. This phenomenon could be attributed to the fact that when the SR content is low, sufficient shear, swelling, and aging cause an easier decomposition of SR into toluene-soluble substances, resulting in a reduction in effective SR content. With increasing SR content, the Q value of SR continues to rise as it undergoes further aging. Additionally, with the increase in SR content, the Q value of SR increases as SR continues to be aged. However, when the content of SR exceeds 12%, the increase in the Q value decreased, and it was difficult for SR particles to be fully shear and swell. Therefore, in the process of heating, shearing, or aging, the degree of chain degradation was weakened, and the increase in the Q value was reduced.

### 3.6. Fourier Transform Infrared Spectroscopy (FTIR)

**Figure 11:**
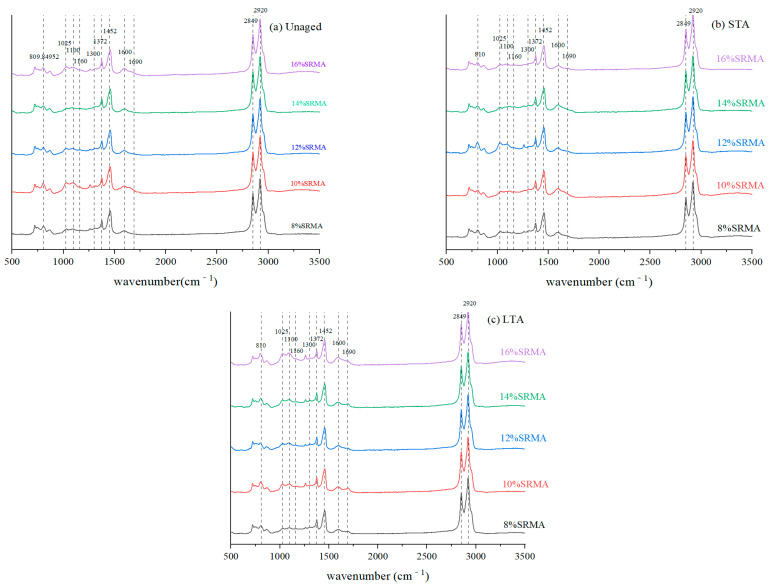
The spectrum of SRMA: (**a**) Unaged; (**b**) STA; (**c**) LTA.

The FTIR of SRMA before and after aging is shown in Figure 11. Obviously, the absorption peaks at 2920 cm^−1^ and 2849 cm^−1^ represented the stretching vibrations of the methylene C-H bonds, and the medium intensity absorption peaks near 1452 cm^−1^ and 1372 cm^−1^ correspond to the flexural vibrations of -CH_2_ and -CH_3_, respectively [39]. In addition, it can be observed that the characteristic peaks of SR mainly include Si-O (800 cm^−1^) in the cross-linking group O-Si (CH)_2_-O, Si-O-Si (1000~1100 cm^−1^) in the main chain of SR [40], and C-H symmetric rocking absorption peak (1260 cm^−1^) in the side chain methyl Si-CH_3_, and the characteristic peak of SR became prominent with the increase of content.

**Figure 12:**
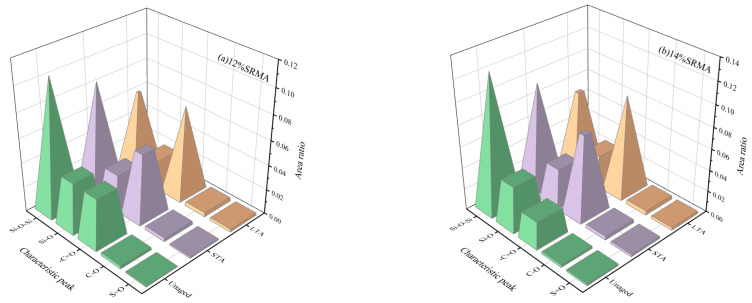
The characteristic peak area ratio of SRMA at different aging stages: (**a**) 12%SRMA; (**b**) 14%SRMA.

Lambert Beer’s law showed that the absorbance of the functional group was proportional to its concentration [41]. Since the total amount of the C-H bond of 2920 cm^−1^ and 2849 cm^−1^ in SRMA changed little during aging, while some characteristic functional groups changed greatly due to aging, the aging degree of SRMA and the degradation degree of SR can be evaluated quantitatively by using the ratio of the characteristic absorption peak of functional groups with greater changes to the characteristic absorption peak of C-H bond with more stable total amount [41]. Because SRMA with different content showed similar laws under different aging degrees, only the infrared characteristic peak area ratio of 12% and 14% SRMA with good performance in the foreword was plotted, as shown in Figure 12a,b. The absorption peak area of Si-O-Si (1000~1100 cm^−1^) representing the main chain of SR and Si-O (800 cm^−1^) in the cross-linking group decreased with the increase in aging degree, indicated that the organic components decreased in terms of both long chain content and cross-linking degree. After SRMA aging, the carbonyl (-C=O) peak at 1600 cm^−1^ and 1690 cm^−1^ increases to a certain extent, the C-O peak at 1025 cm^−1^ becomes prominent, and the content of sulfoxide group (S=O) bond at 1100 cm^−1^ also increases, which was in line with the typical characteristics of asphalt binder aging. In addition, the effect of LTA on functional groups was greater than that of STA. This was due to SRMA’s continuous swelling of rubber in asphalt binder, which was greatly weakened during the LTA of PAV, while its strong oxidation in high-pressure air promoted the occurrence of oxygenation of many asphalt components, which had a much greater impact than the STA of TFOT.

### 3.7. Fluorescence Microscope

**Figure 13:**
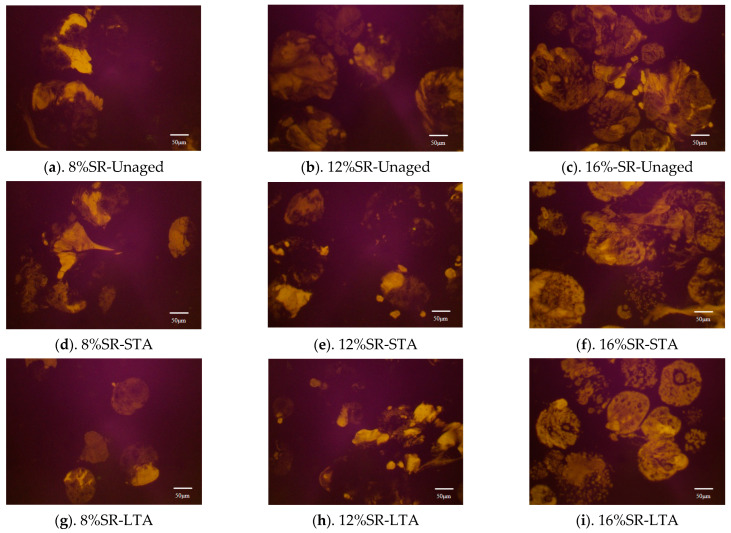
The FM images of SRMA (×400).

To understand the particle shape and dispersion of SR in SRMA before and after aging, it was observed by a fluorescence microscope (FM). The FM images are shown in Figure 13. The dark parts and bright parts each represented the asphalt binder and SR, respectively. When the content of SR is 8%, the dispersion in asphalt is more uniform, and it could fully swell. With the deepening of the aging degree, it can be seen that SR degradation occurred to a certain extent. Furthermore, at the same level of aging, an increase in SR content led to significant expansion of the bright portion, indicating an agglomeration of SR and uneven dispersion of SR particles in asphalt. This is particularly evident when the content reached 16%, as shown in Figure 13, where a large number of SR particles were observed to be agglomerated, leading to incomplete swelling of SR. Under the same content, after STA and LTA of SRMA, SR degradation will greatly reduce its relative molecular weight, resulting in the reduction of the area of the white part. This phenomenon became more obvious with the increase in content.

## 4. Conclusions

This study investigated the rheological properties and chemical composition of SRMA with different contents under different aging degrees. The main conclusions are as follows:

(1) SR could significantly improve the aging resistance and fatigue life of asphalt binder and improve the rutting resistance after aging. SR possesses excellent elasticity and weather resistance. When SR is dispersed within asphalt and fully swollen, it is capable of increasing the elastic components of asphalt and enhancing the resistance of asphalt to high-temperature deformation. When the content of SR attains 12%, SR can effectively shield the aging effect of oxygen on asphalt, thereby improving the anti-aging performance and fatigue life of SRMA.
(2) Adding SR makes the elastic components and the viscosity of the asphalt binder increase, but excessive content was not conducive to the improvement of the high-temperature performance of the asphalt binder. It was speculated that there is an optimal content between 12% and 14%.
(3) SR and asphalt binder will be aged and degraded together in the aging process, but silicone rubber particles had a certain oxygen barrier protection effect relative to asphalt binder in the STA process, and the degradation was obvious in the LTA process.

In this study, the rheological properties, microstructure, and chemical properties of asphalt before and after aging were investigated. On this basis, the influence of waste rubber on asphalt properties in different service states can be further considered. In addition, future research can focus on the compatibility between silicone rubber and asphalt and improvement methods.

## Figures and Tables

**Table 1:** Physical properties of 90#Donghai asphalt.

Items	Technical Indexes	Results	Standard
Penetration (25 °C, 0.1 mm)	80~100	98.0	T 0604-2011
Softening point (°C)	≥44	48.0	T 0606-2011
Ductility (15 °C, cm)	≥100	≥100	T 0605-2011
Ductility (5 °C, cm)	-	43.1	T 0605-2011
60 °C Dynamic viscosity (Pa·s)	≥140	170.35	T 0620-2011

**Table 2:** Physical properties of SR.

Property	Density (kg/m^3^)	Tensile Strength (N/cm)	Elongation at Break (%)	Tear Strength (N/cm)	Hardness (Shore A)
Test value	1.15	83	815	47	61

## Data Availability

Data will be made available on request.
